# Small-World Brain Network and Dynamic Functional Distribution in Patients with Subcortical Vascular Cognitive Impairment

**DOI:** 10.1371/journal.pone.0131893

**Published:** 2015-07-01

**Authors:** Yongqiang Yu, Xia Zhou, Haibao Wang, Xiaopeng Hu, Xiaoqun Zhu, Liyan Xu, Chao Zhang, Zhongwu Sun

**Affiliations:** 1 Department of Radiology, the First Affiliated Hospital of Anhui Medicial University, Anhui, China; 2 Department of Neurology, the First Affiliated Hospital of Anhui Medicial University, Anhui, China; Leibniz Institute for Neurobiology, GERMANY

## Abstract

To investigate the topological properties of the functional connectivity and their relationships with cognition impairment in subcortical vascular cognitive impairment (SVCI) patients, resting-state fMRI and graph theory approaches were employed in 23 SVCI patients and 20 healthy controls. Functional connectivity between 90 brain regions was estimated using bivariate correlation analysis and thresholded to construct a set of undirected graphs. Moreover, all of them were subjected to a battery of cognitive assessment, and the correlations between graph metrics and cognitive performance were further analyzed. Our results are as follows: functional brain networks of both SVCI patients and controls showed small-world attributes over a range of thresholds(0.15≤sparsity≤0.40). However, global topological organization of the functional brain networks in SVCI was significantly disrupted, as indicated by reduced global and local efficiency, clustering coefficients and increased characteristic path lengths relative to normal subjects. The decreased activity areas in SVCI predominantly targeted in the frontal-temporal lobes, while subcortical regions showed increased topological properties, which are suspected to compensate for the inefficiency of the functional network. We also demonstrated that altered brain network properties in SVCI are closely correlated with general cognitive and praxis dysfunction. The disruption of whole-brain topological organization of the functional connectome provides insight into the functional changes in the human brain in SVCI.

## Introduction

Vascular cognitive impairment (VCI) includes all levels of cognitive loss from mild deficits in one or more cognitive domains to a broad dementia syndrome due to cerebral vessel disease [1]. Vascular dementia is currently the second most common type of dementia after Alzheimer’s disease (AD) and seriously affects the patient’s quality of life. Subcortical vascular cognitive impairment (SVCI) is one of the most common subtypes of VCI due to subcortical cerebrovascular disease. It is characterized by extensive white matter hyperintensities (WMH) and multiple lacunar infarctions on magnetic resonance imaging (MRI)[2, 3]. The primary clinical manifestation of SVCI is subcortical syndrome, particularly the executive dysfunction, resulting from the interruption of prefrontal-subcortical loops[4]. Additionally, SVCI is also claimed to induce an increased risk of stroke, dementia, and death[5]. Because of its common occurrence, cost, and possible preventability, SVCI remains of great interest to clinicians and researchers.

Over the past a few years, several techniques such as the electroencephalograph (EEG)[6],diffusion tensor imaging (DTI)[7, 8] and functional MRI(fMRI)[9, 10] have been used to investigate the structural and functional brain changes in patients with VCI/SVCI. In DTI studies[7, 8], SVCI patients exhibited widely disrupted white matter tracts related to cognitive performance. By combining voxel-based morphometry (VBM) and resting-state function MRI(rsfMRI), Yi *et al*.[9]found that the decreased spontaneous low-frequency oscillations (LFO) regions were mainly located in the anterior default-mode network (DMN), while increased low-frequency oscillations regions were located in the posterior DMN in patients with subcortical vascular mild cognitive impairment(svMCI). The above studies helped us to understand the changes of structural and functional brain changes in SVCI patients; however, all of those studies just focused only on local brain regions. Therefore further studies to investigate the functional organization of the entire brain as a network are required.

The human brain is a complex network of spatially distributed but functionally linked brain regions. Recent advances in graph theoretical approaches allow us to characterize the topological properties of complex networks[11]. The small-world network, which is characterized by high a clustering coefficient(Cp) and short characteristic path length(Lp) has both high efficiency of local segregation and global integration of information transmission with low “cost”[12, 13]. Small-world properties are disrupted in several neuropsychological diseases, including in AD[14], Parkinson’s disease(PD)[15] and schizophrenia[16].

However, it remains unclear whether functional alteration of the specific region or inter-regional coordination abnormality occurs in SVCI patients and if it is related to behavioral and cognitive alterations. Here, we hypothesized that SVCI patients may present with abnormalities in the topological properties of brain network connectivity, such as small-worldness and efficiency. To characterize the functional organization of the brain in SVCI, we used graph-analytical methods to compute these network measurements of functional brain connectivity and investigated the relationship between topological properties and cognitive performance.

## Materials and Methods

### Ethics Statement

All research procedures were approved by the Institutional Review Board of the First Affiliated Hospital of Anhui Medical University Subcommittee on Human Studies. Each subject in this manuscript gave written informed consent for participation in this study and publication of these case details. Because cognitive disability can make it impossible to obtain valid informed consent, we also acquired written informed consent from the patients’ caretakers.

### Subjects

Initially, we recruited 27 right-handed patients with SVCI and 22 healthy right-handed age-matched subjects from the first affiliated hospital of Anhui Medical University served as controls. During the research, 3 subjects with SVCI were excluded because they were unable to undergo MRI scanning. In the next preprocessing, 2 controls and 1 SVCI patient were excluded because of head motion. Thus, 23 SVCI patients and 20 controls were used in the final analysis. Patients with SVCI met the following criteria[17]: (1) a subjective cognitive complaint from the patient or his/her caregiver; and (2) a subcortical vascular feature, including a focal neurological symptom or any suggestive sign of cerebrovascular disease or significant white matter hyperintensities (WMH)/lacunar infarcts on MRI scans. Controls matched with SVCI patients for age, sex, and education level were come from either the spouses of patients or recruited via advertisement. They denied any significant cognitive complaints or neuropsychiatric disease or taking any psychoactive medicines. Additionally, they were required to have a mini-mental state examination (MMSE) score of 27 or more based on an evaluation with at least 5 years of education. The details on the diagnosis of SVCI are shown in the diagnosis criteria about the subjects in S1 File.

### Cognitive and Neuropsychological Assessment

A neuropsychological assessment was performed within one week of the MRI scan. All subjects completed a formal neuropsychological assessment administered by a trained neuropsychological technician. A neuropsychological battery was performed, including mini-mental state examination (MMSE), Montreal Cognitive Assessment (MoCA), CAMCOG-C (Cambridge cognitive examination-Chinese version), immediate/delayed word recall, Stroop test, global deterioration scale (GDS), Activities of Daily Living Scale (ADL) and Clinical dementia rating(CDR) to evaluate the function of episodic memory, attention, psychomotor speed, executive function, visuospatial skills and emotion, respectively. The administration of the battery took between 2 and 2.5 hours.

### Neuroanatomical and Resting-State fMRI Data Acquisition and Preprocessing

Functional imaging data were obtained using a 3.0 Tesla GE Signa HDxt MRI scanner (GE, Milwaukee, WI, USA).WMH were assessed using the Fazekas scale[18] (none, punctuate, early con-fluent, confluent; score 0–3) on the fluid-attenuated inversion recovery (FLAIR) sequence. Lacunar infarctions were defined as deep lesions from 3 to 15 mm with CSF-like signal on FLAIR, T1-weighted, and T2-weighted images. All resting-state fMRI data preprocessing was carried out using statistical parametric mapping (SPM8, www.fil.ion.ucl.ac.uk/spm) and Data Processing Assistant for Resting-State fMRI (DPARSF)[19]. For each run, the first 10 volumes of the scanning session were discarded to allow for T1 equilibration effects. Initially, 2 controls and 1 SVCI patients were excluded because of head motion exceeding 3.0 mm movement or 3° rotation in any direction throughout the course of the scan. The remaining 230 volumes were corrected for acquisition delay between slices and realigned to the first volume for head-motion correction. Then, the image data were further normalized to the standard Montreal Neurological Institute (MNI) space and resampled to 3×3×3 mm^3^. Subsequently, the images were spatially smoothed with a 6× 6×6 mm^3^ full width at half maximum Gaussian kernel to minimize spatial noise. Finally, a temporal band-pass filter (0.01< f <0.08 HZ) was employed to remove the effects of low-frequency drift and high-frequency noise.To explain possible anatomical difference, normalized mean gray matter (GM) volume maps of all subjects were calculated using voxel-based morphometry (VBM) toolbox[20]. Details are shown in resting-state fMRI data acquisition and preprocessing in S1 File.

### Network Construction and Graph Theoretical Analysis of Network Connection

Using an automated anatomical labeling template (AAL)[21], fMRI data were registered with the MNI template and further segmented into 90 regions as network nodes. Then, the interregional correlation matrix of the functional connectivity network was defined as a 90×90 undirected graph for each subjects by calculating Pearson’s correlation coefficients between the averaged time series of each possible pair of 90 regions. To determine the threshold T, network sparsity, S_thr_ was applied to each adjacent matrix[22]. We constructed individual brain networks over a wide range of network density, 0.05 ≤ S_thr_ ≤ 0.4, with an increment of 0.01, where the small-world metrics were analyzed[23]. To characterize the topological properties of SVCI and control groups, we also calculated the following network/nodal parameters: clustering coefficient, characteristic path length, global efficiency (E_glob_), and local efficiency (E_loc_)[24]. Both Cp and Lp were the important parameters to quantify the small-world properties of a network in network analysis. Another integrated measure, efficiency, has some advantages over Cp and Lp, as it describes the network behavior at the both global and local levels and constructs either disconnected or nonsparse graphs[24].(see network construction and the topological metrics calculation in S1 and S2 Files for details)

### Statistical Analysis

Statistical comparisons of topological measures between the two groups were performed using a two-sample two-tailed t-test for each value over a wide range of connection densities [0.05–0.4] (P<0.05). If any statistically significant change was found, the distribution of the brain regions was investigated. The area under the curve (AUC) was used to conduct the group comparisons of the metric over the threshold in the GAT toolbox[25]. False discovery rate (FDR) was performed to correct the multiple comparisons for the p value. The x^2^ test was used to compare the differences in sex between the two groups. Pearson’s correlation was applied to analyze the relationship between network or node parameters. (see supplementary materials in S1 File for details).

## Results

### Demographic and Clinical Characteristics of the Participants

As shown in Table 1, there were no significant differences in age, gender and years of education between the two groups, while the SVCI patients showed significantly lower global cognitive scores indicated by MMSE, MoCA, CAMCOG-C and CDR. The higher ADL scores in the SVCI group further indicate the dysfunction in daily living compared to controls. In the subtypes of cognition, SVCI patients had lower scores in word immediate recall, indicating the deficit in both memory and executive/attention function. Moreover, the defect in Stroop performance and the subtypes of CAMCOG-C (praxis, attention) further supported the dysfunction in executive/attention function of SVCI patients.

**Table 1:** Demographic and neuropsychological data.

		SVCI (n = 23)	Controls (n = 20)	*P*-value
Gender (Male%)		55	43.5	0.547^a^
Age (Years)		69.7±8.45	68.75±7.43	0.701^b^
Years of education		8.17±2.44	9.55±2.20	0.060^b^
MMSE		23.35±4.38	28.10±0.97	<0.001^b^
MoCA		18.87±5.11	24.65±1.84	<0.001^b^
CAMCOG-C		75.91±8.39	93.00±4.05	<0.001^b^
	Orientation	8.70±1.61	9.90±0.31	<0.001^b^
	Language	22.30±2.89	27.15±1.73	<0.001^b^
	Memory	19.09±3.66	22.10±2.22	0.003^b^
	Attention	4.65±1.67	6.50±0.76	<0.001^b^
	Praxis	7.52±1.62	10.95±1.00	<0.001^b^
	Calculation	1.83±0.39	2.00±0.00	0.052^b^
	Abstraction	5.29±1.32	6.51±0.76	0.010^b^
	Perception	6.83±1.34	7.09±1.33	0.012^b^
ADL		24.57±9.40	20.25±0.72	0.047^b^
GDS		5.30±2.84	3.95±2.80	0.124^b^
CDR		0.717±0.47	0	<0.001^b^
word-immediate recall		3.74±0.86	4.55±1.54	0.036^b^
word-delayed recall		4.04±1.22	4.85±1.57	0.065^b^
Stroop (dot)		37.56±13.49	23.45±9.75	<0.001^b^
Stroop (characters)		47.86±23.29	31.05±9.61	0.050^b^
Stroop (color)		68.65±25.34	45.60±11.41	0.001^b^
MRI characteristics,NO.(%)				
	Lacunar infarctions present	17(74)	8(40)	0.030^a^
	WMH>1	23(100)	10(50)	<0.001^a^

Data are presented as mean ±SD. MMSE = Mini-Mental State Examination. CAMCOG-C = Cambridge cognitive examination-Chinese version;ADL = Activities of Daily Living Scale; GDS = Global deterioration scale; MoCA = Montreal Cognitive Assessment, CDR = Clinical Dementia Rating; AVLT = Auditory-Verbal Learning Test;WMH = white matter hyperintensities

^a^ two-tail Pearson chi-square test

^b^ were obtained using a two-sample two-tail t-test.

### Global Topological Organization of the Functional Connectome and the Gray Matter Volume

**Fig 1:**
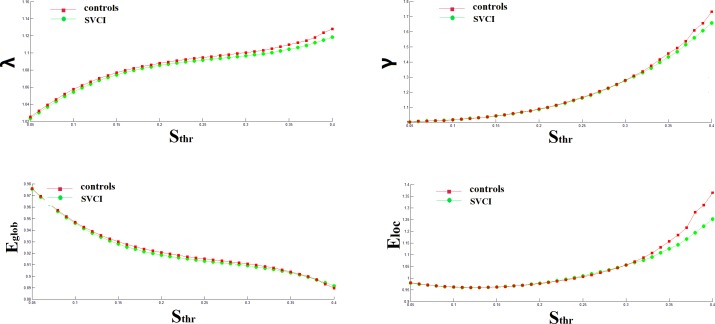
The mean global small-world network metrics of brain networks in healthy controls (red) and patients SVCI (green) as a function of sparsity threshold. λ = normalized shortest path length, γ = normalized clustering coefficient, E_glob_ = global efficiency, E_loc_ = local efficiency.

In this study, we examined the small-world attributes of the resting state network in SVCI patients and matched normal controls. When the sparsity increased, both γ and λ increased in the two networks. Due to the large variance, there were no statistically significant differences in the values of γ or λ between the two groups when the same threshold was used[0.05–0.4] (Fig 1). In range of sparsity [0.15–0.40], all networks in both groups demonstrated small-world architectures compared with the matched random networks, with almost identical characteristic path lengths (λ ≈ 1), but they were more locally clustered (γ > 1) in line with previous rsfMRI studies. Additionally, no significant difference was found in the mean gray matter volume between the SVCI (0.55±0.06 m^3^) and controls(0.57±0.04 m^3^) using the two-sample t-test(t = -1.436, P = 0.159). Further study of single-region gray matter volume yielded comparable results between the two groups.

### Altered Topological Properties of Functional Networks in SVCI Patients

In the range of 0.05 ≤ S_thr_ ≤ 0.40, with increasing connection density, clustering coefficient, global efficiency and local efficiency all increased, whereas characteristic path length decreased in both the SVCI and controls (Fig 1). In the entire threshold range, the characteristic path length of the SVCI group was significantly greater than in the controls, but the clustering coefficient of SVCI group was significantly lower than that of the controls in this range (P<0.05, FDR corrected). In the majority range of threshold, 0.05≤Sthr≤0.36, the global efficiency E_glob_ was significantly decreased in the SVCI group, while the local efficiency E_loc_ was slightly significantly decreased, only in the range of threshold, 0.20≤Sthr≤0.40 (Table 2).

**Table 2:** Significant differences in all metrics between SVIC patients and controls in the range of 0.05 ≤ S_thr_ ≤ 0.40.

Metrics		Regions	Side	t-value	*P*-value
Lp					
	Decreased	Olfactory cortex	L	-2.143	0.0339
		Putamen	L	-2.247	0.0139
		Pallidum	L	-2.345	0.0279
	Increased	Middle temporal gyrus	L	3.114	0.0199
		DCG	R	2.174	0.0236
Cp					
	Decreased	Supplementary motor area	R	-2.744	0.0226
		DCG	L	-3.484	0.0012
		DCG	R	-3.493	0.0136
		Postcentral gyrus	R	-2.148	0.0342
		Precuneus	R	-2.525	0.0317
		Middle temporal gyrus	L	-3.784	0.0117
E_glob_					
	Decreased	DCG	L	-2.214	0.0428
		DCG	R	-2.745	0.0254
		Superior temporal gyrus	R	-3.173	0.0317
		Temporal pole: superior temporal gyrus	L	-2.268	0.0287
		Middle temporal gyrus	L	-2.393	0.0214
	Increased	Putamen	L	2.369	0.0294
		Pallidum	L	2.419	0.0095
E_loc_					
	Decreased	Supplementary motor area	R	-2.027	0.0482
		DCG	L	-3.707	0.0045
		Inferior occipital gyrus	R	-1.785	0.0114
		Paracentral lobule	L	-4.745	0.0089
		Superior temporal gyrus	L	-2.166	0.0462
	Increased	Olfactory cortex	L	2.13	0.0485

t, statistical value showing nodal topological properties difference (P < 0.05,FDR corrected) between two groups (positive t-value means increased topological properties in the SVCI group); DCG: median cingulate and paracingulate gyri; R, right; L, left.

### Distribution of the altered regions in the brain

**Fig 2:**
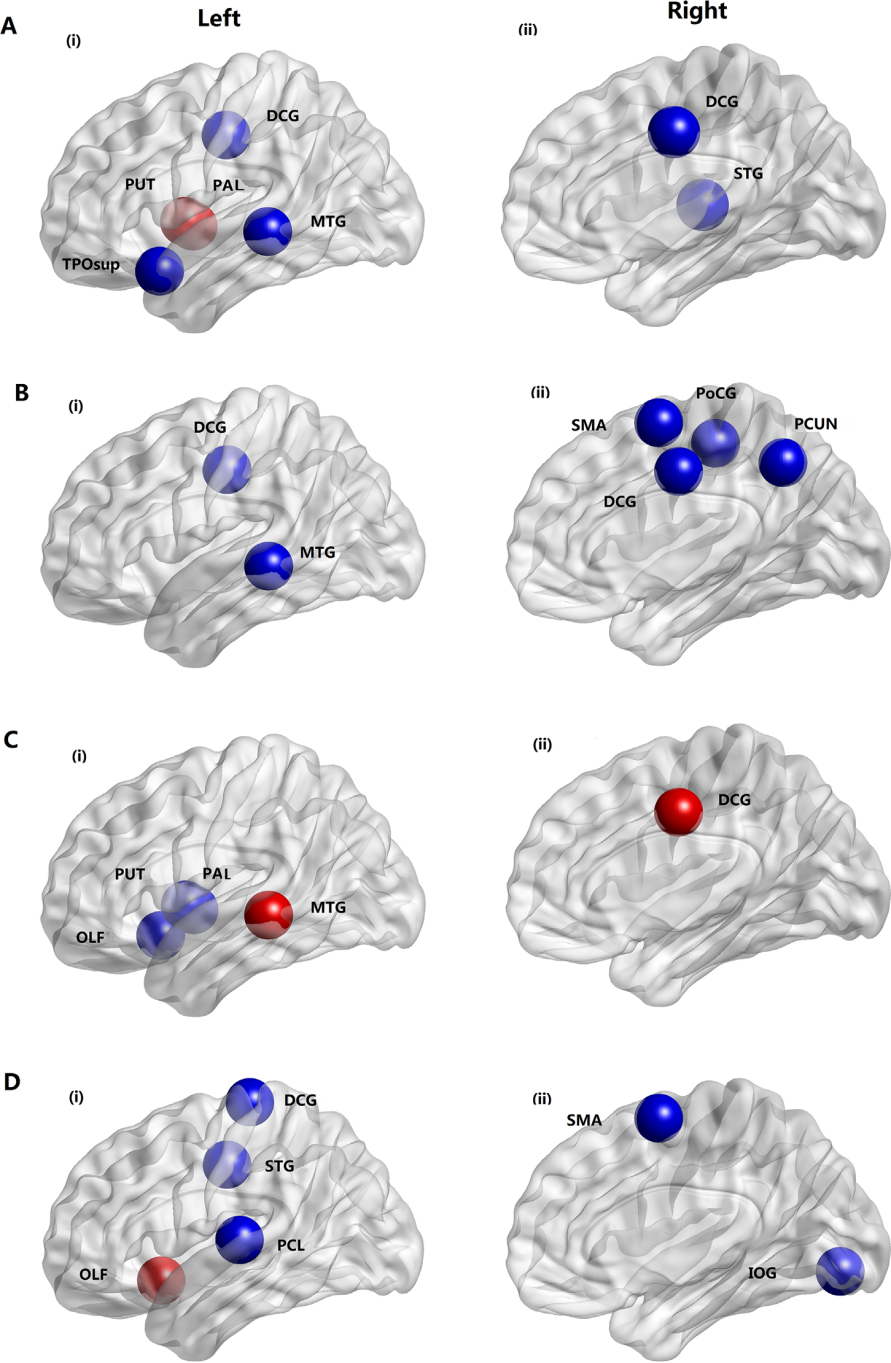
Distribution of brain regions in which small-world properties decreased(blue) or increased(red) significantly in SVCI patients compared to the healthy controls over a wide range of correlation thresholds (0.05<T<0.4). (A) global efficiency; (B) clustering coefficient; (C) characteristic path length; (D) local efficiency; DCG = median cingulate and paracingulate gyri; MTG = middle temporal gyrus; PUT = putamen; PAL = pallidum; TPOsup = temporalpole: superior temporal gyrus; STG = superior temporal gyrus; SMA = supplementary motor area; PCUN = Precuneus; PoCG = postcentral gyrus; OLF = Olfactory cortex; PCL = paracentral lobule; IOG = Inferior occipital gyrus

Two-sample t-tests for each of the 90 regions were performed to further localize the nodes that demonstrated significant differences between SVCI and normal control groups. Since all thresholds exhibited a similar trend, we have chosen a typical threshold (T = 0.18) to report. Fig 2 and Table 2, show the regions with significant alterations in the clustering coefficient and local efficiency that were widely distributed across the brain. The clustering coefficient in many regions was decreased in SVCI, such as the median cingulate and paracingulate gyri (DCG), supplementary motor area (SMA), left middle temporal gyrus (MTG.L), postcentral gyrus(PoCG) and right precuneus(PCUN.R). The local efficiency was decreased in both regions of the frontal-temporal lobe and parietal lobe in SVCI, such as DCG, SMA, right inferior occipital gyrus (IOG.R), left paracentral lobule (PCL.L) and left superior temporal gyrus (STG.L), while the local efficiency was increased in the olfactory cortex in SVCI.The regions showing significant alterations in global efficiency and characteristic path length were also distributed primarily in the frontal-temporal lobe, such as the DCG, middle temporal gyrus and regions in the subcortical structure, such as the left lenticular nucleus pallidum (PAL.L) and left lenticular nucleus putamen (PUT.L).

### Relationships between Topological Measurements and Cognitive Performance in SVCI Patients

**Fig 3:**
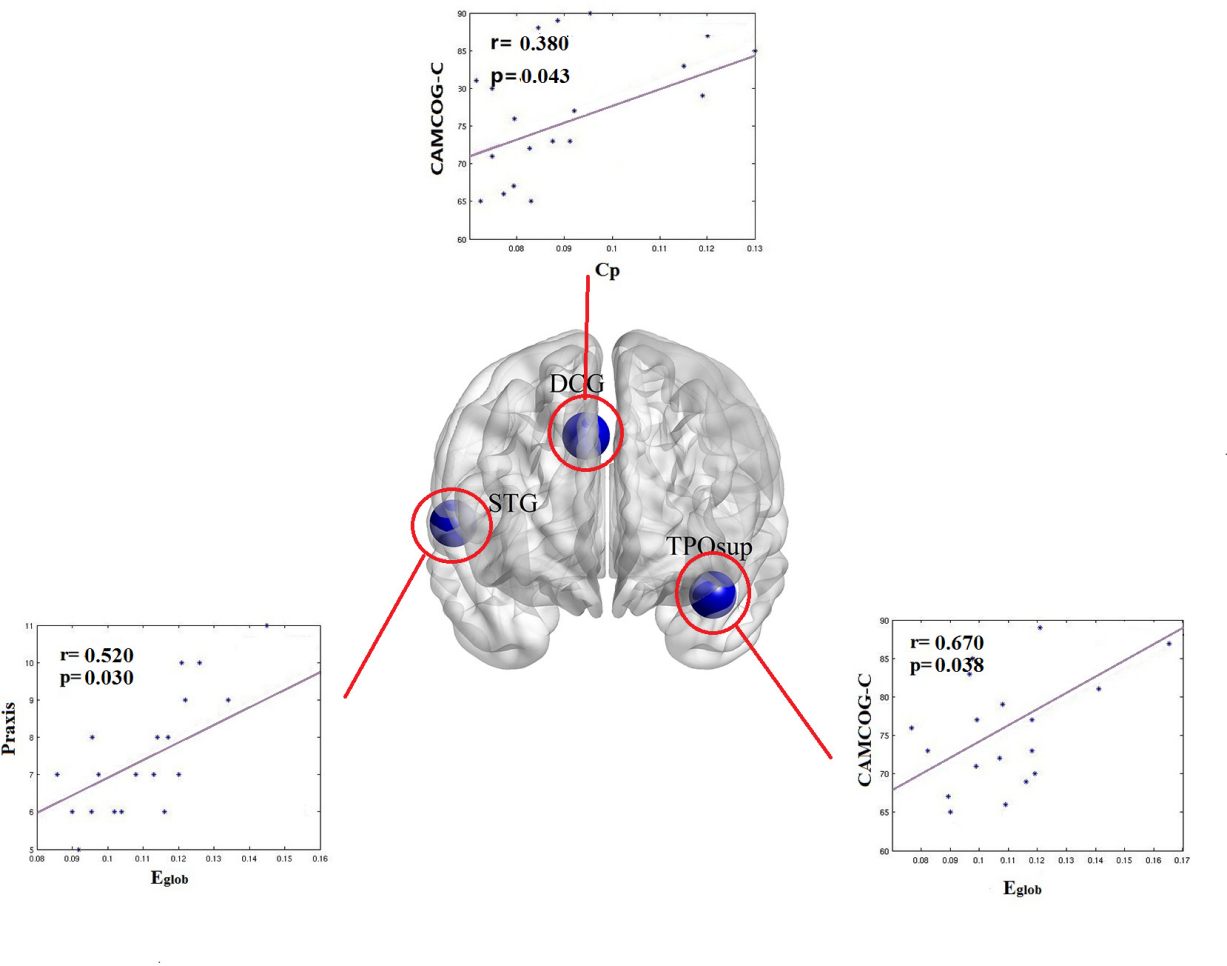
The relationship between global network/nodal characteristics and CAMCOG-C scores/ of the praxis functional score patients with SVCI. CAMCOG-C:Cambridge cognitive examination-Chinese version. Cp = clustering coefficient; E_glob_ = global efficiency.

Significant (P<0.05, corrected) correlations between topological metrics and CAMCOG-C total scores in SVCI patients were found in the clustering coefficient and global efficiency as a function of sparsity(0.08–0.31). In the DCG, the whole network clustering coefficient was positively correlated with CAMCOG-C total scores. Additionally, global efficiency distributed in the temporal pole also exhibited a positive correlation with the sum scores of CAMCOG-C. For the subscale of CAMCOG-C, global efficiency distributed in the right superior temporal gyrus was positively correlated with the praxis (Fig 3).

## Discussion

Using graph-analytical methods, we found that the functional brain networks of both groups exhibited small-world organization patterns. However, compared to controls, the SVCI group showed disturbed global/local graph metrics. Overall, the regions of decreased efficiency were primarily located in the fronto-temporal and the parietal lobes, while the increased region was mainly located in the subcortical area, which is compatible with the disturbed frontal-subcortical loops in patients revealed by a previous study[3]. Additionally, our study showed that the abnormal global topological organization of functional networks was correlated with cognitive performance. This suggested that altered topological properties may be responsible for behavioral and cognitive deficits in SVCI patients.

It is widely known that, leukoaraiosis is commonly detected by brain MRI in aging individuals as white matter hyperintensities, and has been considered to present an increased risk of stroke and to serve as an important predictor of the cognitive impairment and functional disability[5]. In normal controls, leukoaraiosis could be commonly observed due to the vascular factors and normal aging, while the grade of leukoaraiosis was mostly mild compared to that of the SVCI patients. In the current study, the leukoaraiosis was exhibited in both SVCI patients and a portion of the controls. The different regions between the two groups, as shown in the extracted-overlay images were similarly located in the frontal-temporal lobe, which was generally consistent with the abnormal node in the aforementioned network analysis in SVCI patients. These findings might give us a cue that the dysfunctions in ischemic regions largely influence the efficiency of information transfer and communication, as indicated by the disconnection or inefficiency in our graph theory analysis.

In the present study, functional brain networks of both SVCI and control subjects showed small-world attributes over a range of sparsity thresholds (0.15–0.400)[24, 26, 27]. These properties enable high efficiency of both segregated and integrated information processing at a relatively low wiring cost across the whole brain, which allows the brain to resiliently face pathological attacks, including ischemic lesion caused by arteriosclerosis[28–31]. However, over the range of 0.05–0.15, no small-world attributes were exhibited in the groups, which was inconsistent with previous studies[16, 32]. One main possibility is the methodological differences in the data analyses. For example, different methods (EEG or resting-state fMRI) applied into subjects and different corrections were carried out. In our manuscript, we found that after correction the groups had a lack of significant small-world properties at lower sparsity thresholds (0.05<sparsity<0.15). Another important possible reason is the difference between the subjects involved in those studies and in our manuscript. From a clinical perspective, schizophrenia and SVCI exhibit different behavioral symptoms and imaging findings with different underlying neural mechanisms. This may also be the critical factor that affects the results.

In spite of small-world attributes in both groups, graph theoretical metrics were significantly altered in patients with SVCI. Over nearly the entire range of the sparsity threshold, the nodal clustering coefficient was significantly decreased in SVCI patients when compared to controls. Since the clustering coefficient denotes the extent of local cliquishness, the decreased clustering coefficient may imply relatively lower local connectivity of functional networks, and inefficiency in information transfer for cognitive processes between interconnected regions[28, 31]. In the present study, the decreased clustering coefficient was widely distributed across the brain, and mainly located in the frontal-temporal lobe.These findings are consistent with previous structural studies, which identified decreased cortical thinning in frontal and temporal cortices in patients with SVCI[33]. The defects in the frontal-temporal cortices are primarily correlated with the executive/attention abilities, accompanied with visual memory and visuomotor skills. The decrease in the clustering coefficient could be attributable to the disorders of inter-regional functional connectivity and imply brain functional connectivity disorders within and between regions.

A short characteristic path length implies the effective integrity and rapid information propagation between and across remote regions of the brain, which is believed to constitute the basis of cognitive processing[31]. In contrast, long characteristic path length may reflect disrupted neuronal integration between the regions. In our study, between-group comparisons revealed that the brain structures were targeted predominantly in the frontal-temporal lobe and the subcortical structure, which was largely in line with the regions associated with the frontal-subcortical loops. Additionally, largely coinciding with the clustering coefficient decreases, network efficiency analysis revealed significantly decreased local efficiency and global efficiency in part of the range in SVCI patients. A previous study showed that the global efficiency is affected by the loss of long-range connections[26]. Structural and DTI studies[7, 8] found that some fronto-temporal lobes and occipital regions, which were associated with long fibers, were abnormal in SVCI patients, incurring disruption to long-range communication across the brain, ultimately impacting cognition in SVCI patients. The above structural abnormalities may be attributable to the decreased tendency of global efficiency of brain networks in SVCI. Given the correlation of structural abnormality with the cognition or functional network, we hypothesized that alterations in the efficiency of the network might be related to the cognitive and behavioral alterations correspondingly. In the current study, part of the significantly increased efficiency in the brain networks was also found in SVCI patients. The underlying mechanisms of increased local efficiency of a network are still under investigation. A previous study indicated that a network with higher efficiency might have larger fault tolerance to face the external attack[24]. In a spinal cord injury study, the increase in local efficiency was suggested to contribute to functional reorganization or plasticity[34]. Thus, the higher values of efficiency in SVCI patients observed here might indicate a defense mechanism responsible for resisting pathological attacks. Taken together, our results revealed that the brain networks in SVCI patients were disrupted, reflecting a less optimal topological organization, thereby providing further evidence of cognitive dysfunction in SVCI patients. In addition, to shed some light on the relationship between functional and structural alterations, we carried out an additional investigation. The fact that no significant difference was found in mean gray matter volume between SVCI and controls, despite the functional topological differences, indicates that the functional abnormalities may occur prior to structural atrophy.

To further evaluate the relationship between the network/nodal metrics and cognition, the CAMCOG-C score was entered into the analysis as an important variable. The CAMCOG-C score, a part of the CAMDEX[35], has a high test-retest reliability to evaluate the general level of cognitive function as well as the subtype cognition, as characterized by its subitems assessing orientation, attention, praxis, and so on[36]. In the present study, we found that the whole network clustering coefficient was positively correlated with the CAMCOG-C total scores in DCG. This finding further supported the previous network research in which a network with a high clustering coefficient will accelerate the information communication and transfer, which are the basis of cognition. Additionally, the global efficiency distributed in the region of temporal pole also exhibited a positive correlation with the sum scores of CAMCOG-C. This result also indicated that the higher global efficiency, the higher information interactions among brain components. In the analysis of the subtype cognition, the global efficiency also showed a positive relationship with the praxis in the right superior temporal gyrus. In a previous study[37], the superior temporal gyrus was not so much focused on its role in language but in processing social stimuli and was reported to play a prominent role in the monitoring and reappraisal of behavior. Additionally in Asaf Achiron’s study[38], the thickness of the superior temporal gyrus was else closely related to the cognitive performance in aspects of attention and information processing speed. These findings might indicate the important roles of the superior temporal gyrus in the process of the vascular cognition impairment and add to the understanding of the neural basis of SVCI.

The present study strongly suggested that SVCI patients are characterized by a disrupted functional and structural integrity in multiple distributed neuronal networks as well as in whole brain systems. However, several limitations in this study should be considered. First, though we have adopted advanced graph analysis, there are still some limitations of the methodology and materials. For instance, similar to most resting-state fMRI studies, the effects of physiologic noise can not be eliminated in our study, although a band-pass filtering of 0.01–0.08 Hz was used to reduce physiological noise. Second, considering the small sample size, the difference between mild and severe cognition impairment levels of SVCI patients has not been compared. Future study will be required to investigate the topological properties in the mild SVCI and subcortical vascular dementia(SVaD) group separately to better understand the development of SVCI. Finally, this is the first exploratory study of SVCI patients using graph-theoretical analyses of resting-state fMRI data; therefore, further research must be conducted to confirm these findings.

## Conclusions

In summary, similar to normal controls, SVCI patients also exhibited small-world attributes but topological abnormalities in the whole brain network were evident. The altered globe/nodal properties were widely located in the frontal-temporal lobe and subcortical region, which is consistent with the regions in front-subcortical loops. This graph theoretical study improves our understanding of the mechanism of cognitive dysfunction in SVCI patients and may guide the early diagnosis and even the treatment of SVCI in the future.

## Supporting Information

S1 File Contains details of diagnosis criteria about the subjects, resting-state fMRI data acquisition and preprocessing, network construction, topological metrics calculation and statistical analysis. (PDF)

S2 File All of the preliminary data after preprocessing and network analysis underlying the findings. (RAR)
