# Supplementary material for: Small-World Brain Network and Dynamic Functional Distribution in Patients with Subcortical Vascular Cognitive Impairment
Source: PLoS One. 2015 Jul 1;10(7):e0131893. doi: 10.1371/journal.pone.0131893 (PMC4489389; doi:10.1371/journal.pone.0131893)
Supplement: S1 File — (PDF) [file pone.0131893.s001.pdf]

**Small-World brain network and dynamic functional distribution in patients with subcortical vascular cognitive impairment**

**Diagnosis Criteria about the Subjects**

## Subjects

Twenty-seven right-handed subjects with SVCI and twenty-two healthy right-handed age-matched subjects were enrolled from the first affiliated hospital of Anhui Medical University. Prior to the experiment, the purpose of the study was briefly explained to the subjects. The diagnosis of SVCI was made by experienced neurologists and should meet the following criteria[1]: 1) subjective cognitive complaints reported by the participant or his/her caregiver; 2) objective cognitive impairments in any aspects including memory, language, executive, attention, or visuospatial based on neuropsychological test; 3) any subcortical vascular feature associated with a focal neurologic symptom or suggestive sign that included corticobulbar signs, pyramidal signs, or parkinsonism, such as gait disorder, urgent urination, motor slowness, dysarthria, lower facial weakness, or sensory deficit, and 4) magnetic resonance imaging (MRI) exhibited significant ischemia, including lacunar infarcts or significant white matter hyperintensities(WMH) on their MRI scans, which was defined as a cap or band >10 mm and a deep white matter lesion >25 mm modified from the ischemia criteria described in a previous study [2]. SVCI patients were further classified subcortical vascular mild cognitive impairment (svMCI) and dementia (SVaD).

SVaD patients had more severe cognitive impairment and further met the diagnostic criteria: 1) a Clinical Dementia Rating Scale (CDR) score >0.5; 2) objective cognitive impairments meeting the Diagnostic and Statistical Manual of Mental Disorders, fourth edition (DSM-V) criteria for dementia [3]. In current study, 27 SVCI patients were subtyped as the 20 SMCI patients and 7 SVaD patients.

Participants were excluded if they had any of the following clinical characteristics: with a history of acute stroke, head injury, Parkinson's disease, epilepsy, major depression, alcoholism,or other neuropsychiatric illness, and patients with severe visual or hearing loss, dentures or metallic stent in vivo, who were unable to complete the MRI scanning and

assessment.

Additionally, the comparison of medical histories between the two groups was shown in table 1.

Table 1 Baseline clinical characteristics of the subjects (n (%)

| | SVCI (n=23) | Controls (n=20) | *P*-value |
|---|---|---|---|
| Hypertension[a], no.(%) | 13(56.5) | 8(40.0) | 0.364 |
| Diabetes Mellitus [b], no.(%) | 6(26.17) | 4(20.0) | 0.728 |
| Current smoker, no.(%) | 3(13.0) | 4(20.0) | 0.687 |
| Alcohol consumptiom[c], no.(%) | 5(21.7) | 6(30.0) | 0.728 |
| Use of anti-platelet aggregation medication, no.(%) | 12(52.2) | 8(40) | 0.544 |
| Use of blood pressure-lowering medication , no.(%) | 13(56.5) | 8(13.4) | 0.364 |
| Use of plasma glucose-lowering medication, no.(%) | 6(26.1) | 4(20.0) | 0.728 |
| Use of lipid-lowering medication, no.(%) | 10(43.5) | 6(30.0) | 0.528 |

**Resting State fMRI Data Acquisition and Preprocessing**

Here we provide a detailed description of the fMRI data acquisition and preprocessing steps. Functional imaging data were performed using a 3.0 Tesla GE Signa HDxt MRI scanner (GE, Milwaukee, WI, USA).).A Three-dimensional T1-weighted images was acquired with the following parameters: TR=9.5 ms; TE=3.9 ms; TI=450 ms; flip angle=$20^0$; field of view=256 mm; matrix size=512×512. The resting-state data were obtained using echo-planar imaging (EPI). After structural MRI scans, resting-state fMRI scans were acquired. During the resting scan, the participants were instructed to relax, keep their eyes closed without falling asleep and not to think anything in particular. The 8-minute resting-state scan was comprised of 240 contiguous echo planar imaging whole brain functional volumes with the following parameters: TR=2 s, TE=30 ms, FOV=240 mm, flip angle $80^0$, matrix size 64 × 64, thickness=4mm, gap=0.6mm.

All resting state fMRI data preprocessing was carried out using statistical parametric mapping (SPM8, http:// www.fil.ion.ucl.ac.uk/spm) and Data Processing Assistant for Resting-State fMRI (DPARSF)[4].

## Neuroanatomical data preprocessing

To examine or evaluate the potential effects of vascular lesions independently to relation of the network metrics and cognition, the visible lesion of each subjects' T2-fluid-attenuated inversion recovery (FLAIR) images were overlapped manually slices by slices according to SVCI group and controls separately. To inspect the difference between the two groups in morphology, the overlapped images in controls was subtracted from the ones in SVCI group to obtain discrepant images. The ischemia regions in extracted-overlay images were considered as the target area of vascular factors to the SVCI patients approximately. Because of the manual tracing technique, no automated spatial normalization was necessary. Lesion maps were resampled led to a l-mm isotropic voxel size, smoothed with a Gaussian kernel (4mm full width a thalf maximum), and binarized by using a threshold of 0.2 (http://www. mccauslandcenter.sc.edu/mricro/mricro/overlay/index.html).

**Network Construction and Topological Metrics Calculation**

### Network construction

To define the network node, the functional images were registered with the standard Montreal Neurological Institute (MNI) template and further divided into 90 regions using an automated anatomical labeling template(AAL) [6], which has been used in several previous studies[7, 8]. The mean time series from each of the 90 regions were calculated by averaging the time series of all of the voxels within that region. To reduce the effects of physiological processes, average signals from the global brain, white matter (WM), cerebral spinal fluid (CSF), gray matter (GM), along with the motion parameters(3 rotational and 3 translation) were regressed out and removed from the data. Then, regression residuals were substituted for

the raw mean time series of the corresponding region. Next, the interregional correlation matrix of functional connectivity network was defined as a 90×90 undirected graph for each subjects by calculating Pearson's correlation coefficients between the averaged time series of each possible pair of 90 regions.. Then, a Fisher r-to-z transformation was adopted to improve the normality of the partial correlation coefficient and construct a binary network. If the absolute z (i,j) (Fisher r-to-z of the partial correlation coefficient) of between a pair of brain regions, i and j, exceeds a given threshold T, an edge was assumed to exist; otherwise it does not exist. To determine the threshold T, the network sparsity,($S_{thr}$) ,which was defined as the actual number edges in a graph divided by the maximum possible number of edges, was applied to each adjacent matrix [9]. Using sparsity-specific threshold enables that the resulting networks of both groups have the same density level. Given the fact that the selection of $S_{thr}$ is critical to the topological metrics of networks, we constructed the individual brain networks over a wide range of network density, $0.05 \leq S_{thr} \leq 0.4$, with an increment of 0.01, where the small-world metrics are analyzed [10]. Sparse networks of each subject were constructed using a minimum spanning tree method followed by global thresholds.

## Gray Matter volumes

On T1-weighted images, normalized mean gray matter (GM) volumes were calculated using the Structural Imaging Evaluation of Normalized Atrophy (SIENAx) software.[11] Global GM volumes, including the 90 AAL cortical and subcortical regions were measured using voxel-based morphometry (VBM) and the Diffeomorphic Anatomical Registration using Exponentiated Lie algebra (DARTEL) registration method [12] in SPM8. First, individual anatomical images were classified into gray matter, white matter and cerebrospinal fluid as well as three extra-cerebral tissue classes. Then, GM maps were normalized to the GM population-specific template generated from the complete image set using DARTEL [12]. Spatially normalized images were then modulated by multiplying with the Jacobian determinants derived from the spatial normalization. Then, the AAL template was resampled in the DARTEL space. Global GM volumes of each subject were calculated as the mean value

of all the voxels within the all regions. To further study the difference of single region gray matter volume between the two groups, the GM map of each subject was finally smoothed with an 8-mm full-width at half maximum kernel and put into the building general linear model(GLM).To test the result, on T1-weighted images, normalized mean gray matter (GM) volumes were also calculated using the Structural Imaging Evaluation of Normalized Atrophy (SIENAx) software.[12]

## Small-world network

The clustering coefficient C is equal to the number of connections of a node with its nearest nodes proportional to the maximum of possible neighboring connections. The characteristic path length L is corresponding to the average number of minimum connections that are required to join any two nodes [7, 13]. Networks having small-worldness properties are defined as those significantly more clustered than random networks but having approximately the same characteristic path length as random networks. Thus, normalized clustering coefficient and characteristic path length are obtained: $\gamma = Cp / C_{random}$ and $\lambda = Lp / L_{random}$. The ratio $\sigma = \gamma/\lambda$ is often used and must be >1 ($\gamma > 1$ and $\lambda \approx 1$) to define the small-worldness of a network [14, 15].The Brain Connectivity Toolbox was used to construct the random network at different threshold[16]. For comparison, 50 random networks were computed with the same number of nodes, total edges,and degree of the original network[17].

## Efficiency

Global efficiency is a measure of the network's capacity for parallel information transfer between nodes via multiple series of edges. There is strong evidence that the brain supports massively parallel information processing; therefore, it is conceptually preferable to adopt efficiency metrics of brain functional network topology.

Global efficiency is inversely related to the "classical "small-world metric of average minimum path length. Thus, a small-world network will have global efficiency greater than a regular lattice but global efficiency less than a random network. The global efficiency of a

graph G is calculated as[13, 15]:

$$E_{glob} = \frac{1}{N(N-1)} \sum_{j \neq i G} \frac{1}{L_{ij}} .$$

Where $L_{i,j}$ is the shortest path length between regions i and j.The path length between regions i and j is defined as the sum of the edge lengths along this path, while the shortest path length $L_{i,j}$ between regions i and j is the path length with the shortest length between the two regions.

The local efficiency of the graph $G_{i,}$ a subgraph of whole graph G, is the average of the local efficiency of all of its nodes in this subgraph. It can measure how efficient the information is transferred in the subgraph.and be defined as [15]:

$$E_{loc}(G) = \frac{1}{N} \sum_{i \notin G} E_{glob}(G_i) .$$

**Statistical analysis**

To compare the topological properties (clustering coefficient, characteristic path length, local efficiency, global efficiency) throughout the preselected sparsity threshold between the two groups, a two-sample t-test was performed. We selected a range of sparsity thresholds, $0.05 \leq S_{thr} \leq 0.4$, with an increment of 0.01.The area under the curve (AUC) was used to conduct the group comparisons of the metric over the threshold in the GAT toolbox[18].The false discovery rate (FDR) correction was applied for multiple comparisons and the threshold restricting the expected proportion of type I errors to lower than 0.05 was estimated. The nodes with significantly different metrics (P<0.05, FDR corrected) were chosen as region of interests. These regions of interests were displayed by using BrainNet Viewer (Version 1.0 RC1, http://www.nitrc.org/projects/bnv/). Pearson's correlation was applied to analyze the relationship between network or node parameters (clustering coefficient, characteristic path length, global efficiency, local efficiency) and cognitive scores in the SVCI group. A P-value<0.05 after correction for multiple comparisons by using FDR was considered to indicate a significant difference.

**References:**


1. Park JH, Seo SW, Kim C, Kim SH, Kim GH, et al.(2014) Effects of cerebrovascular disease and amyloid beta burden on cognition in subjects with subcortical vascular cognitive impairment. Neurobiology Of Aging 35: 254-260.
2. Fazekas F, Schmidt R, Scheltens P(1998)Pathophysiologic mechanisms in the development of age-related white matter changes of the brain. Dement Geriatr Cogn Disord 9 Suppl 1: 2-5.
3. Rockwood K, Howard K, MacKnight C, Darvesh S(1999) Spectrum of disease in vascular cognitive impairment. Neuroepidemiology 18: 248-254.
4. Chao-Gan Y, Yu-Feng Z(2010) DPARSF: A MATLAB Toolbox for "Pipeline" Data Analysis of Resting-State fMRI. Front Syst Neurosci 4: 13.
5. Birn RM, Murphy K, Bandettini PA(2008)The effect of respiration variations on independent component analysis results of resting state functional connectivity. Human Brain Mapping 29: 740-750.
6. Tzourio-Mazoyer N, Landeau B, Papathanassiou D,Crivello F,Etard O,et al.(2002) Automated anatomical labeling of activations in SPM using a macroscopic anatomical parcellation of the MNI MRI single-subject brain. Neuroimage 15: 273-289.
7. Zhao X, Liu Y, Wang X, Liu B,Xi Q, et al.(2012) Disrupted small-world brain networks in moderate Alzheimer's disease: a resting-state FMRI study. PLoS One 7: e33540.
8. Supekar K, Menon V, Rubin D, Musen M, Greicius MD, et al. (2008) Network analysis of intrinsic functional brain connectivity in Alzheimer's disease. PLoS Comput Biol4: e1000100.
9. Qian S, Sun G, Jiang Q, Liu K, Li B, et al.(2013) Altered topological patterns of large-scale brain functional networks during passive hyperthermia. Brain Cogn 83: 121-131.
10. Watts DJ, Strogatz SH(1998) Collective dynamics of 'small-world' networks. Nature 393: 440-442.
11. Smith SM, De Stefano N, Jenkinson M, Matthews PM(2001) Normalized accurate measurement of longitudinal brain change. J Comput Assist Tomogr 25:466-475.
12. Ashburner, J(2007) A fast diffeomorphic image registration algorithm. NeuroImage 38,

95–113.

13. Achard S, Bullmore E(2007) Efficiency and cost of economical brain functional networks. PLoS Comput Biol 3: e17.
14. Sporns O, Zwi JD(2004) The small world of the cerebral cortex. Neuroinformatics 2: 145-162.
15. Latora V, Marchiori M(2001)Efficient behavior of small-world networks. Physical Review Letters 87: 198701.
16. Rubinov M, Sporns O (2010) Complex network measures of brain connectivity: uses and interpretations. Neuroimage 52: 1059–1069.
17. Maslov S, Sneppen K (2002) Specificity and stability in topology of protein networks. Science 296: 910–913.
18. Hosseini SM, Hoeft F, Kesler SR (2012) GAT: a graph theoretical analysis toolbox for analyzing between-group differences in large-scale structural and functional brain networks. PLoS ONE 7:e4070.
